# Seven Years Cognitive Functioning and Early Assessment in Extremely Low Birth Weight Children

**DOI:** 10.3389/fpsyg.2017.01257

**Published:** 2017-07-21

**Authors:** Chiara Squarza, Odoardo Picciolini, Laura Gardon, Maura Ravasi, Maria L. Giannì, Matteo Porro, Matteo Bonzini, Silvana Gangi, Fabio Mosca

**Affiliations:** ^1^Neonatal Intensive Care Unit, Department of Clinical Sciences and Community Health, Fondazione IRCCS Ca’ Granda Ospedale Maggiore Policlinico, Università degli Studi di Milano Milan, Italy; ^2^Protection and Promotion of Workers Health Unit, Department of Clinical Sciences and Community Health, Fondazione IRCCS Ca’ Granda Ospedale Maggiore Policlinico, Università degli Studi di Milano Milan, Italy

**Keywords:** extremely low birth weight, school outcome, early assessment, Griffiths Scales, cognitive functioning

## Abstract

Infants born preterm are at high risk for the onset of cognitive dysfunctions at school age. The aim of this study was to investigate the association between early neurodevelopmental assessment and the risk of adverse cognitive outcome in extremely low birth weight children. We enrolled all newborns (January 2002 – April 2007) consecutively admitted to our Institution, with a birthweight < 1000 g. Exclusion criteria were genetic abnormalities, severe neurofunctional impairment, and/or neurosensory disabilities. Ninety-nine children were assessed at 1 year of corrected age using the Griffiths Mental Development Scales Revised. The same children were re-assessed at school age through the Wechsler Intelligence Scale for Children. Children with impaired Griffiths General Quotient (i.e., <1 SD) at 1 year of corrected age showed a significantly lower Full Scale Intelligence Quotient at 7 years of chronological age when compared to children who scored in the normal range at 1 year (*p* < 0.01). Considering the Griffiths Sub-quotients separately, a poor score in the Performance or in the Personal-Social Sub-quotients at 1 year was associated with significantly worse cognitive outcomes both in the Verbal and in the Performance Intelligence Quotients at 7 years (*p* < 0.01 and *p* < 0.05, respectively). A score <1 SD in the Locomotor or in the Eye and Hand Coordination Sub-quotients were specifically associated with poorer Performance or Verbal Intelligence Quotients, respectively (*p* < 0.05). Our findings suggest that a poor score on the Griffiths Scales at 1 year is associated with a higher risk of cognitive impairment at school age. Larger confirmation studies are needed.

## Introduction

Improvements in medical knowledge and techniques for high-risk infants have progressively reduced the rates of mortality and major sequelae in preterm infants, especially with ELBW (<1000 g) (Doyle et al., 2011; Latini et al., 2013). At the same time, an increased risk for long-term minor neurobehavioral and cognitive deficits has been reported (Marlow et al., 2005).

Very preterm birth (i.e., born < 32 weeks’ gestational age) and ELBW are most often co-occurring conditions.

Very preterm infants with ELBW may experience a disruption of important processes involved in early brain development, as the last trimester of pregnancy is an essential period for the creation and organization of neuronal connections, underlying thinking, learning, and feeling (deRegnier, 2008).

Pioneering brain-scanning studies support the idea that altered networks play a part in cognitive problems of preterm and ELBW infants. Hüppi (2010) found that, compared with children born at term, the premature children’s neuronal tracts were organized less efficiently, often taking a more meandering path. These changes in organization were correlated with reduced social and cognitive skills.

In addition, the premature birth triggers an emotional crisis in parents, mainly as a result of their concern for the infant’s survival, the frequent invasive treatment in the NICU, and the protracted separation from the child. The alteration of the parents’ role can affect the way they perceive their child and respond to him (Holditch-Davis and Miles, 1997; Allen et al., 2004).

Several studies suggest that the challenge of premature birth struggles the maternal responsiveness, thus reducing social skills and cognitive development among preterm ELBW children (Landry et al., 1997). The lengthy hospital stay and the separation from their parents hinders the parent–infant bond, reducing infant self-regulation (Bhutta et al., 2002; Clark et al., 2008; Wolke et al., 2014).

These factors must be taken into account as they might have damaging repercussions on the global development of the preterm ELBW infant.

Longitudinal studies demonstrate that children born extremely preterm and/or ELBW are at increased risk for neurobehavioral impairments, including cognitive deficits, learning disabilities, and behavioral and emotional problems at school age (Hack et al., 2009; Anderson and Doyle, 2003). Difficulties in these areas have been related with academic struggles and higher rates of special education support (Msall, 2012). Indeed, self-regulation is generally considered an integrated ability of cognitive skills, that reflects functional performance (Howe et al., 2016).

An estimated 40–70% of preterm ELBW children have been identified with minor impairments, such as borderline-to-low average IQ, mild motor problems, and poor adaptive behavior during preschool and school years (Allen, 2008), while the expected rates of mild impairment in a population of normal birth weight children would be around 16%.

A complex set of factors have been associated to developmental outcomes of preterm ELBW children. Among the strongest predictors of later cognitive outcome for preterm ELBW children without severe disabilities, medical complications, maternal education, and early developmental assessment are often mentioned. The influence of perinatal risk factors tends to decrease over time as environmental factors become more important (Linsell et al., 2015).

The identification of early developmental markers that may be predictive of long term cognitive outcome is essential for prevention and rehabilitation (Orton et al., 2009). However, few studies have addressed the association between early neurodevelopmental quotients and cognitive outcomes at school age.

In this context, our study focuses on the association between multiple neurodevelopmental skills assessed at 1 year and separate domains of intelligence at 7 years, thus suggesting some insight about the complex integration of specific developmental abilities and their longitudinal association.

In current literature, there is mixed evidence about the predictive validity of the Griffiths Mental Developmental Scales (Griffiths and Huntley, 1996), a neurodevelopmental assessment tool commonly administered in clinical and research practice. Sutcliffe et al. (2010), considering a sample of infants born at term and with normal birth-weight, reported that the Griffiths Scales, completed at 17 months, had a significant predictive power for Performance IQ and Full Scale IQ but not for the Verbal IQ at age 5. Other studies suggest that the General Quotients obtained at 2 and 3 years of age with the Griffiths Scales strongly correlate with intellectual ability at 5 years assessed through the Stanford Binet in a cohort of ELBW infants (Bowen et al., 1996). The validity of the Griffiths Scales is also suggested by its strong agreement with the Bayley Scales (Picciolini et al., 2015).

The aim of this study was to investigate the extent to which Griffiths’ neurodevelopmental quotients at 1 year of corrected age are associated with cognitive functioning at 7 years of chronological age in a cohort of children born very preterm with ELBW.

We speculate that the deprivation of a critical period of rapid intrauterine growth, the altered neuronal connections and the clinical, emotional, and environmental challenges cited above (in particular, the lack of social and motor experiences due to the lengthy hospitalization and the protracted separation from parents) might significantly influence the early neurodevelopmental pathways of premature infants with ELBW. In this perspective, we hypothesize that motor and emotional skills could be the most affected domains during the first year of life.

Secondly given the deep integration among intellectual and neuropsychological skills in early infancy, we hypothesize that these early impairments should have long term effects on the cognitive maturation of these high-risk infants.

## Materials and Methods

We performed a single-center longitudinal cohort study. The study design was approved by the Ethics Committee of the Fondazione IRCCS Ca’ Granda Ospedale Maggiore Policlinico and written informed consent was obtained from all the infants’ parents.

Inclusion criteria were having a weight < 1000 g and a gestational age < 32 weeks at birth. Exclusion criteria were the multiple birth, the presence of genetic abnormalities, severe neurofunctional impairment (defined as Neurofunctional Assessment > 2, Picciolini et al., 2006; Gianní et al., 2007), and/or neurosensory disabilities (blindness, deafness). We decided such exclusion criteria because having fine motor control and sensory orientation was a prerequisite for undergoing the cognitive assessment we administered.

All the ELBW babies admitted to NICU were enrolled after discharge in the follow-up program provided at authors’ Institution and were scheduled to be prospectively followed up to 7 years of chronological age. Infants were enrolled in the study at 1 year of corrected age in occasion of the neurodevelopmental assessment and were re-assessed at 7 years to evaluate their cognitive outcome.

All the children of our sample underwent the same follow-up procedures, as part of a standard follow-up program. Children with severe neurofunctional impairment at 1 year of corrected age were enrolled in specific rehabilitation programs (i.e., neuromotor developmental therapy, and/or neuropsychological interventions), while children with milder signs of impairment were accurately observed step by step along with the follow-up visits, activating a parental counseling and creating a clinical network with local pediatricians.

A number of neonatal characteristics, including gender, birth weight, being adequate or small for gestational age, mode of delivery, multiple birth, duration of hospital stay, were collected from the infants’ computerized medical charts. Gestational age was based on the last menstrual period and early ultrasound examination; infants with birth weight ≥ 10th centile or < 10th centile for gestational age, according to the Fenton Growth Chart (Fenton, 2003), were classified, respectively, as AGA/SGA. The occurrence of IVH grade 3 or higher, according to the Papile classification scheme (Papile et al., 1978), PVL of grade 2 or higher, according to de Vries et al. (1992), ROP of stage 3 or higher, according to the International Classification of Retinopathy of Prematurity (Committee for the Classification of Retinopathy of Prematurity, 1984), and BPD, defined as oxygen supplementation at 36 weeks postmenstrual age (Jobe and Bancalari, 2001), were also collected. IVH and PVL were detected by brain MRI examination at 40 weeks postmenstrual age. Corrected age was calculated up to 24 months of life, from the chronological age adjusting for gestational age. Mothers’ nationality and education were also recorded. Mothers’ educational level was used as a measure of socioeconomic status and classified using a 3-point scale, where 1 indicates primary or intermediate school education (≤8 years), 2 secondary school education (9–13 years), and 3 university degree (>13 years).

### Neurodevelopmental Assessment

According to our Follow-up program, neurodevelopmental outcome at 1 year was assessed using the validated Italian translation of the Griffiths Mental Development Scales Revised (Battaglia and Savoini, 2007), administered by two trained examiners. This tool comprises a set of five subscales, specifically investigating neurodevelopment in the Locomotor, Personal-Social, Hearing and Language, Eye and Hand Coordination and Performance domains. The Scale yields standardized Sub-quotients for each domain (mean 100, SD 16) and a composite General Quotient (mean 100, SD 12). In accordance with this, children were classified as having typical development if their General Quotient was 88 or more and their Sub-quotients were 84 or more and they were classified as having a developmental delay if their General Quotient was 87 or lower and their Sub-quotients were 83 or lower. Because normative data of the Griffiths Mental Development Scales Revised are not available in our country, we referred to the 1996 United Kingdom norms (Griffiths and Huntley, 1996).

### Cognitive Assessment

The cognitive functioning at 7 years of age was assessed by the Italian version of the WISC-III (Wechsler, 1991). This test consists of 13 subtests that are combined into three IQ scores (mean 100, SD 15); Full Scale IQ, Verbal IQ, and Performance IQ. Accordingly, children were classified as having a normal intelligence if their IQ scores were 85 or more while they were classified as having a developmental delay if their IQ scores were 84 or lower. The cognitive testing was performed by a specially trained psychologist, blind to the child’s performance at the Griffiths Scales.

### Statistical Analyses

Neurodevelopmental and cognitive results for children, respectively, at 1 year and 7 years, were summarized by mean, SD, and range. For each scale we identified low performing children when score was lower than 1 SD below the mean. Dividing low performing and normal performing children at the Griffiths Scales, we compared mean values of IQ scores at 7 years, testing differences with Student’s *t-*test. Then, we designed a multivariate logistic regression model, including potential confounders (selected *a priori* based on literature search, in details: gender, AGA/SGA, BPD, MRI, and maternal education), to calculate relative risk (expressed as adjusted ORs) of resulting low performing at WISC-III IQ scores after scoring low performing at the Griffiths Scales at 1 year.

A *p*-value < 0.05 was considered as statistically significant and relative CIs at 95% of confidence were calculated for all ORs. All data analyses were performed using software Stata (Stata Corp, Austin, TX, United States).

## Results

**FIGURE 1:**
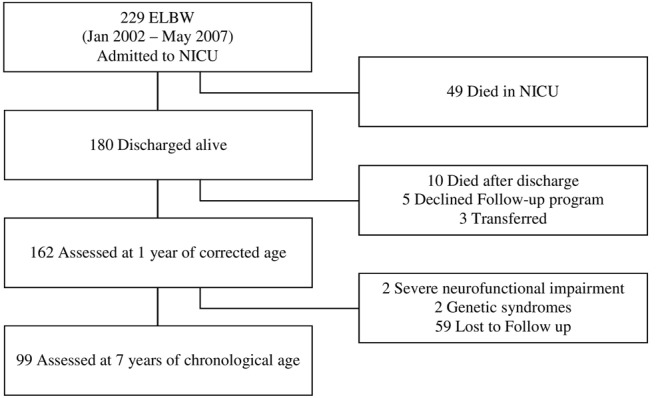
Flow chart of the study.

A total of 229 very preterm ELBW infants were admitted to NICU Fondazione IRCCS Ca’ Granda Ospedale Maggiore Policlinico, between January 2002 and April 2007. Among them, 180 (79%) were discharged home alive and enrolled in the follow-up program provided by the Authors’ Institution. Of these, 162 (90%) returned for the 1-year follow-up visit and 99 (55%) were assessed at 7 years and entered the study. No differences in the neonatal and growth variables were observed between infants who were lost to follow up and those who were evaluated. The flow chart of the study is shown in **Figure 1**.

The mean gestational age at birth for our very preterm ELBW cohort was 27.7 weeks (SD: 2.3) and mean birth weight was 769.7 g (SD: 165.5). Of the 99 infants enrolled, 43 (43%) were males, 58 (59%) were SGA and 15 (15%) were multiple births. 88 (90%) infants were born by cesarean delivery and mean hospitalization was 97.1 days (SD: 32.7). BPD was diagnosed for 42 (42%) infants and grade III/IV ROP for 10 (10%) infants. 6 (6%) infants had PVL, while 5 (5%) had IVH grade III/IV. Mothers’ mean age was 33.7 years (SD: 4.5), 56 of them (57%) had a high school education and 20 (20%) a university degree.

The mean Griffiths General Quotient and Sub-quotients at 1 year of corrected age and mean WISC-III IQs at 7 years of chronological age were in the average range (**Table 1**). The Griffiths Locomotor Sub-quotient had the lowest mean score. No differences were found between participants and dropouts considering the mean Griffiths General Quotient and Sub-quotients.

**Table 1:** Griffiths and WISC-III mean quotients at 1 and 7 years.

*N* = 99	Mean (*SD*)	Range	Number (%) < 1 SD	Number (%) < 2 SD
**Griffiths Scales 1 year**	
General Quotient	94.2 (9.5)	65-113	21 (21.2)	4 (4.1)
Locomotor	88.5 (11.1)	58-108	29 (29.3)	6 (6.1)
Personal-Social	92.7 (10.5)	60-113	17 (17.2)	3 (3.0)
Language	98.5 (9.2)	78-122	6 (6.1)	–
Eye and Hand Coordination	92.9 (10.8)	60-117	20 (20.2)	1 (1.0)
Performance	94.2 (12.5)	55-118	17 (17.2)	3 (3.0)
**WISC-III 7 years**				
Full Scale IQ	103.0 (15.7)	52-139	11 (11.1)	3 (3.0)
Verbal IQ	104.8 (15.9)	51-137	10 (10.1)	4 (4.0)
Performance IQ	100.3 (14.4)	62-148	10 (10.1)	2 (2.0)

As displayed in **Table 2**, children who gained a score < 1 SD at the Griffiths General Quotient at 1 year of corrected age showed a significantly lower Full Scale IQ at 7 years of chronological age than children who scored in the normal range at 1 year (*p* < 0.01).

**Table 2:** Differences on mean IQs at 7 years based on neurodevelopmental assessment at 1 year.

Griffiths Scales 1 year (*N* = 99)		WISC-III 7 years (*N* = 99)			
		Full Scale IQ			
		Mean *(SD)*		*p*-value	
General Quotient *normal range*	*n* = 78	105.6 (14.1)		0.005	
General Quotient <* 1 SD*	*n* = 21	95.0 (17.5)			

		**Verbal IQ**		**Performance IQ**	
		**Mean *(SD)***	***p*-value**	**Mean *(SD)***	***p*-value**

Locomotor *normal range*	*n* = 70	106.8 (13.4)	0.114	102.7 (12.0)	0.024
Locomotor <* 1 SD*	*n* = 29	101.3 (19.8)		95.6 (18.0)	
Personal-Social *normal range*	*n* = 82	107.0 (14.3)	0.010	101.9 (13.7)	0.044
Personal-Social <* 1 SD*	*n* = 17	96.4 (19.2)		94.2 (15.7)	
Language *normal range*	*n* = 93	105.7 (15.0)	0.171	101.2 (13.9)	0.116
Language <* 1 SD*	*n* = 6	96.7 (24.3)		91.7 (19.2)	
Eye–Hand Coordination *normal range*	*n* = 79	107.1 (14.1)	0.018	101.9 (13.4)	0.067
Eye–Hand Coordination <* 1 SD*	*n* = 20	97.9 (19.5)		95.4 (16.8)	
Performance *normal range*	*n* = 82	107.7 (13.0)	0.000	102.4 (12.8)	0.004
Performance <* 1 SD*	*n* = 17	93.3 (21.5)		91.6 (17.9)	

*Student’s *t-*test on mean WISC-III IQs at 7 years, comparing low performing (scores < 1 SD) and normal performing (scores in the normal range) children at the Griffiths Scales at 1 year.*

Considering the Griffiths Sub-quotients separately, a score < 1 SD in the Personal-Social or in the Performance Sub-quotients at 1 year was associated with significantly worse cognitive outcomes both in the Verbal and in the Performance IQs at 7 years (*p* < 0.05 and *p* < 0.01). A score < 1 SD in the Locomotor or in the Eye and Hand Coordination Sub-quotients at 1 year was specifically associated with poorer Performance or Verbal IQs, respectively, at 7 years (*p* < 0.05).

Unadjusted and adjusted results from regression analysis are reported in **Tables 3, 4**, respectively. A score < 1 SD at the Griffiths General Quotient at 1 year moderately predicted subsequent impairment on WISC-III Full Scale IQ, after adjustment for a number of biological and social factors (**Table 4**).

**Table 3:** Odds of cognitive impairment at 7 years based on neurodevelopmental assessment at 1 year.

Griffiths Scales 1 year (*N* = 99)	WISC-III 7 years (*N* = 99)			
	Total IQ			
	Odds ratio		95% CI	
General Quotient	4.5		1.16; 17.4	

	**Verbal IQ**		**Performance IQ**	
	**Odds ratio**	**95% CI**	**Odds ratio**	**95% CI**

Locomotor	3.39	0.84; 13.67	5.74	1.33; 24.83
Personal-Social	8.02	1.89; 34.15	2.68	0.59; 11.99
Language	2.1	0.22; 20.25	6.07	0.94; 39.16
Eye and Hand Coordination	10.71	2.39; 47.96	3.65	0.88; 15.13
Performance	27.65	5.03; 151.9	8.02	1.88; 34.15

*Odds ratio of cognitive impairment (<1 SD) at 7 years based on Griffiths General Quotient and subscale scores < 1 SD at 1 year.*

**Table 4:** Odds of cognitive impairment at 7 years based on neurodevelopmental assessment at 1 year after adjustment for potential confounders^∗^.

Griffiths Scales 1 year (*N* = 99)	WISC-III 7 years (*N* = 99)			
	Total IQ			
	Odds ratio		95% CI	
General Quotient	4.24		0.95; 18.99	

	**Verbal IQ**		**Performance IQ**	
	**Odds ratio**	**95% CI**	**Odds ratio**	**95% CI**

Locomotor	2.63	0.49; 14.10	4.26	0.88; 20.59
Personal-Social	7.45	1.29; 42.97	2.38	0.46; 12.12
Language	0.83	0.04; 15.39	4.64	0.62; 34.59
Eye and Hand Coordination	9.22	1.66; 51.22	3.51	0.75; 16.35
Performance	62.30	5.43; 714.58	9.13	1.77; 46.91

*Odds ratio of cognitive impairment (<1 SD) at 7 years based on Griffiths General Quotient and subscale scores < 1 SD at 1 year. ^∗^ Adjusted for gender, AGA/SGA, BPD, MRI 40 weeks, maternal education.*

Three of the Griffiths Sub-quotients were strongly associated with an increased risk of impairment at the WISC-III Verbal IQ, the Performance Sub-quotient (OR 62.30, 95% CI [5.43; 714.58]), the Eye and Hand Coordination Sub-quotient (OR 9.22, 95% CI [1.66; 51.22]) and the Personal-Social Sub-quotient (OR 7.45, 95% CI [1.29; 42.97]), with no prediction from the Language Sub-quotient (OR 0.83, 95% CI [0.04; 15.39]).

Considering the Performance IQ, the Griffiths Performance Sub-quotient (OR 9.13, 95% CI [1.77; 46.91]), the Language Sub-quotient (OR 4.64, 95% CI [0.62; 34.59]) and the Locomotor Sub-quotient (OR 4.26, 95% CI [0.88; 20.59]) were the most powerfully associated.

## Discussion

Our findings suggest that a Griffiths General Quotient < 1 SD at 1 year of corrected age increases the odds of low IQ scores at 7 years by 4.24 (95% CI [0.95; 18.99]), adjusting for biological, neonatal, and family factors. The highest associations were shown between Personal-Social, Eye–Hand Coordination and Performance subscales at 1 year and Verbal IQ at 7 years and between Locomotor, Language, and Performance subscales at 1 year and Performance IQ at 7 years.

Our findings suggest that a score < 1 SD on the Griffiths General Quotient at 1 year of corrected age is associated with a higher risk of impairment on subsequent IQ scores at school age. A low score on the Performance Sub-quotient is the most associated with school-age cognitive impairments, especially for the Verbal IQ. Eye and Hand Coordination, Personal-Social and Locomotor Sub-quotients also show an association with later cognitive functioning.

These results may be explained by the fact that early neurodevelopmental impairments emerging during the first year of life represent the basis for the onset of later cognitive underachievement. Specifically, a difficulty in manipulating materials or in organizing visual-spatial skills, as assessed by the Griffiths Performance subscale, reveal a lack of cognitive flexibility affecting long term intellectual outcome (Squarza et al., 2016).

Actually, the particularly strong association between the Performance subscale at 1 year and the cognitive functioning at 7 years might be explained considering that this subscale specifically focuses on a variety of intellectual skills that are strongly related with later cognitive functioning.

To enhance the maturation of early cognitive skills, motor experiences and activities with objects are essential. Accordingly, a low score on the Locomotor and Eye–Hand Coordination Sub-quotients at 1 year was significantly associated with long term cognitive impairments. Early motor impairments, hindering the child in discovering the environment and taking contact with objects and persons, may interfere with the maturation and organization of higher cognitive functions. Indeed, in literature there is evidence of a strong association between early motor development and later intellectual functions within the normal population (Murray et al., 2006).

At the same time, the association between Eye-Hand Coordination Sub-quotient and Verbal IQ at school age may be related to the fact that an active exploration of objects and activities, such as showing and offering objects to adults, may enable infants to extend periods of social interactions and to enhance the emergence of symbolization abilities. This provides opportunities for infants to expand language and learn rules of interaction, as reported in previous studies (Karasik et al., 2011).

With regard to the Personal-Social domain, the importance of emotional regulation for an adequate cognitive functioning is widely recognized in literature. Follow-up studies confirm that behavioral and social-emotional immaturity may negatively affect cognitive functions and academic achievements in preterm infants (Gianní et al., 2006; Aarnoudse-Moens et al., 2009; Pugliese et al., 2013).

The predictive validity of early developmental assessment for long term cognitive outcomes has been questioned since neurodevelopmental evaluation in the first 2 years of life may not reliably predict the full spectrum of disability at school age, particularly for less severe impairments (Aylward, 2002). According to the EPICURE study (Marlow et al., 2005), very early neurodevelopmental assessments at 6 to 12 months may not accurately predict neurodevelopmental impairment at school age in 25% of children born before 26 weeks of gestational age. Hack et al. (2005) found that infant testing at 20 months of corrected age in ELBW is more predictive among children with severe handicaps and/or subnormal functioning.

In contrast to these findings, in the present study we found a strong association between early developmental assessment and long term cognitive outcome in children with only a mild impairment (<1 SD). The discrepancy between previous findings and ours may be almost partly related to the different assessment tool. Both these studies referred to the Bayley Scales for the early neurodevelopmental assessment. Although the Griffiths Scales have shown a good concordance with the Bayley Scales (Picciolini et al., 2015), they are a more complex and integrated instrument, assessing not only cognition, language, and motor abilities but also personal-social skills, thus providing a more comprehensive evaluation of the child, perhaps more associated with long term outcome.

Consistently with our results, other studies revealed that early neurodevelopmental assessments of preterm infants are good predictors of school readiness. Patrianakos-Hoobler et al. (2010) found that 2-year neurodevelopmental delay (MDI or PDI < 70) predicted the need for special education services at the age of 5 years, while children with MDI or PDI scores lower than 85 (1 SD below the mean) had an increased risk of not being ready for school. Barnett et al. (2004) examined a cohort of term infants with neonatal encephalopathy and found that an abnormal score (<1 SD) on the Griffiths Sub-quotients at 1 and/or 2 years was very likely to be associated with poor performance at school age. Although this study was performed on a cohort of infants born at term, it could yet be considered a population of infants at risk, which is the same target of our study, aiming at identifying early markers of risk for long term cognitive impairment.

Studying a cohort of VLBW infants, Breeman et al. (2015) found that cognitive function in adulthood could be fairly well estimated from age 20 months and that IQ scores were most stable for VLBW individuals who had severe cognitive impairment in adulthood. Breeman et al. (2015) also found that the Griffiths Mental Development Scales did not reliably predict cognitive development at 5 months, probably because of the faster state fluctuation of cognitive function in younger infants. In our study, the association with long term cognitive outcome is found at an earlier age (1 year) and is strong also considering infants with only a mild impairment.

In our study, the rates of ELBW infants with a developmental impairment at 1 year stand between 6.1–23.2% for the 1 SD cut-off and between 0.0–6.1% for the 2 SD cut-off. At 7 years the rates of mild and severe impairment are around 10.1–11.1% and 2.0–4.0%, respectively.

These rates are not higher than those expected from a normal birth weight population (23.7–30.2% and 3.2–4.4%, respectively for the Griffiths Scales at 1 year and around 16% for the WISC-III at 7 years).

In our perspective, this is not surprising given that syndromic and severely impaired children were excluded from our study. Moreover, all the children of our sample were assessed at 1 year through the Neurofunctional Assessment, which has been demonstrated to correlate with later neurodevelopmental outcome, probably limiting the rates of impairment at 1 year and at 7 years in our sample (Picciolini et al., 2016).

However, the particularly low rates of impairment in the Language subscale at 1 year (6.1% < 1 SD) is remarkable. This could be explained considering that at 1 year the early communication skills are still emerging and that specific language delays will arise later, during the second year of life, with the expansion of the vocabulary and the influence of emotional maturation (specifically the separation-individuation process).

Our study has several limitations. The lack of a normal weight control group represents a major issue and prevents from clearly attributing the outcomes observed in our sample to any specificities in the developmental pathways of very preterm ELBW children, or to the developmental trajectories that characterize a normal birth weight population.

Actually, our study shows an association between early neurodevelopmental assessment and the risk of adverse cognitive outcome at 7 years. In particular, our findings suggest specific areas of impairment in the developmental profiles at 1 year that seem very specific in comparison with a normal distribution based on normative data (e.g., locomotor abilities). These findings are particularly useful because the timely identification of neurodevelopmental impairments associated with later cognitive outcome is essential to plan rehabilitative programs.

Further studies including a normal weight control group are required in order to confirm if the neurodevelopmental pathways we observed are preterm and ELBW specific and to verify if the association with long term cognitive outcome could be effective also for the normal population.

Moreover, the relatively small sample size with consequent large uncertainty in our risk estimates (i.e., 95% CI) deserves caution in interpreting our results. Moreover, it is possible that the weak association between the Language subscale at 1 year and the Verbal IQ at 7 years might be partially due to the translation of the Griffiths Scales from the original standardization language to Italian. Another limitation is the relatively high rate of children lost to follow up, which may limit the interpretation of our findings. However, we found no differences in the neonatal and growth variables between infants who were lost to follow up and those who were evaluated.

Future long-term studies will need to include larger populations and to assess not only cognitive functioning, but also behavioral and emotional outcomes. Nevertheless, a strength of our study is that it is a longitudinal study that provides information on the long term cognitive outcome of a population of high risk children. Specifically, our findings strengthen the evidence of a deep integration among cognitive functions, in particular between early motor and later cognitive skills and between verbal and performance domains.

## Conclusion

In summary, our study suggests that early neurodevelopmental assessment is associated with school-age cognitive achievement in a cohort of very preterm ELBW children. According to our findings, even a mild neurodevelopmental impairment at 1 year of corrected age seem to be associated with long term cognitive deficits. These results have important implications for clinical services and follow-up programs since provision of timely intervention is dependent upon accurate, early identification of infants at risk for adverse long-term outcomes. Our findings recommend timely activation of intervention programs so that early impairments at 1 year do not lead to more stable cognitive difficulties at later ages. Based on our findings, we would recommend starting a rehabilitation program for those children who scored < 1 SD at the Griffiths Scales at 1 year.

## Author Contributions

CS, OP, LG, and MR conceptualized and designed the study, drafted the initial manuscript, and approved the final manuscript as submitted. MG, MP, and MB designed the data collection instruments, carried out the initial analyses, reviewed and revised the manuscript, and approved the final manuscript as submitted. FM and SG coordinated and supervised data collection, critically reviewed the manuscript, and approved the final manuscript as submitted. All authors approved the final manuscript as submitted and agree to be accountable for the content of the work.

## Conflict of Interest Statement

The authors declare that the research was conducted in the absence of any commercial or financial relationships that could be construed as a potential conflict of interest.

## Abbreviations

AGA/SGA: adequate/small for gestational age
BPD: bronchopulmonary dysplasia
CI: confidence interval
ELBW: extremely low birth weight
IQ: intelligence quotient
IVH: intraventricular hemorrhage
MRI: magnetic resonance imaging
OR: odds ratio
PVL: periventricular leukomalacia
ROP: retinopathy of prematurity
SD: standard deviation
WISC-III: Wechsler Intelligence Scale for Children Third Edition
